# Meat Attachment and Consumption Patterns in Primary Care: A Cross-Sectional Study

**DOI:** 10.7759/cureus.89457

**Published:** 2025-08-06

**Authors:** Paul Sebo, Bruno Delaunay, Benoit Tudrej, Mohamed Amir Moussa, Hubert Maisonneuve

**Affiliations:** 1 University Institute for Primary Care, University of Geneva, Geneva, CHE; 2 University College of General Medicine, University Claude Bernard Lyon 1, Lyon, FRA

**Keywords:** gender, maq, meat attachment, meat attitude, meat consumption, primary care, switzerland

## Abstract

Background: Meat consumption is deeply embedded in many cultures but poses significant health and environmental challenges. This study investigates the association between attachment to meat, as measured by the validated French Meat Attachment Questionnaire (MAQ), and actual meat consumption among primary care patients.

Methods: This cross-sectional study was conducted in primary care practices in Geneva, Switzerland, from January to May 2024. A total of 425 patients were invited to participate. Participants were non-urgent, French-speaking, consecutive adult patients who were able to understand the study and provide written informed consent, and data were collected using self-administered questionnaires during routine consultations. The French version of the 16-item MAQ (MAQ-16) was used to assess attachment to meat, and meat consumption patterns for poultry, beef, veal, and pork were measured. ANOVA and multivariable linear regressions were conducted to examine associations between meat attachment and consumption.

Results: Of the 425 invited patients, 336 accepted the invitation, resulting in a participation rate of 79%. The sample comprised 61% women, with a median age of 53 years. Participants had a mean MAQ score of 3.3 (SD: 0.7), with male individuals reporting significantly higher scores than female individuals (3.5 vs. 3.2, adjusted difference: 0.4 (95% CI: 0.2-0.6), adjusted p-value<0.001). Poultry was the most frequently consumed meat, with 39% of participants consuming it more than once a week. Men reported higher meat consumption across all types examined. Higher MAQ scores were significantly associated with greater meat consumption; for example, those consuming poultry more than once per week had a mean score of 3.5 compared to 2.7 for non-consumers (adjusted difference: 0.8 (95% CI: 0.6-0.9), adjusted p-value<0.001).

Conclusion: This study demonstrates that a strong emotional attachment to meat is significantly associated with higher levels of meat consumption. Understanding these psychological factors can inform public health strategies aimed at promoting dietary changes, addressing both health outcomes and environmental sustainability.

## Introduction

Meat consumption remains a significant dietary component in many cultures, despite growing awareness of its environmental, ethical, and health-related consequences [1]. While numerous public health initiatives encourage reduced meat consumption and the adoption of plant-based diets, the emotional and psychological attachment to meat represents a key barrier to behavior change [2]. Understanding this attachment is crucial for informing interventions aimed at promoting sustainable dietary practices [2,3] and addressing health outcomes associated with excessive meat consumption [4,5]. The Meat Attachment Questionnaire (MAQ) was developed to assess individuals' attachment to meat across four dimensions: hedonism, affinity, entitlement, and dependence. The tool has been used in various cultural contexts to explore the cognitive and emotional ties people have with meat consumption [6-12] and has been shown to predict willingness and intention to adopt a more plant-based diet [6]. Recently, our research team adapted and validated a 17-item French version of the MAQ (MAQf-17) [12], establishing it as a reliable instrument for assessing meat attachment in French-speaking populations.

However, while the MAQ effectively measures attachment and meat attachment has been shown to be a strong predictor of meat consumption [7,13], little is known about the direct association between the MAQ and actual meat consumption behavior [7]. Identifying whether a strong attachment to meat, as measured by the MAQ, correlates with higher meat consumption could provide valuable insights into dietary habits and inform tailored strategies for reducing meat intake. Exploring this association is particularly relevant in primary care settings, where healthcare professionals are increasingly tasked with addressing the dietary behaviors that contribute to chronic diseases.

The objective of this study was to examine the association between attachment to meat, as measured by the French version of the MAQ, and actual consumption of land-based meats (poultry, beef, veal, and pork) among adult primary care patients in Geneva. By investigating this relationship, we aim to contribute to the validation of the MAQ in French and provide healthcare providers with insights to better understand and address meat consumption behaviors in their patients. Seafood consumption was excluded from the study due to cultural and psychological distinctions that often separate it from land-based meats, ensuring a more focused analysis of attachment to terrestrial meats.

## Materials and methods

Study design and setting

This cross-sectional study was conducted with patients recruited from primary care practices in the canton of Geneva, Switzerland. The study aimed to assess the relationship between meat attachment, as measured by the French version of the MAQ, and meat consumption patterns. The study was conducted in a primary care setting to explore the relationship between meat attachment and meat consumption in a clinically relevant population where dietary habits are frequently discussed. While this population may include individuals with chronic conditions, it also comprises generally healthy adults attending routine consultations. Future research should extend this investigation to broader community-based samples.

Participants

**Figure 1:**
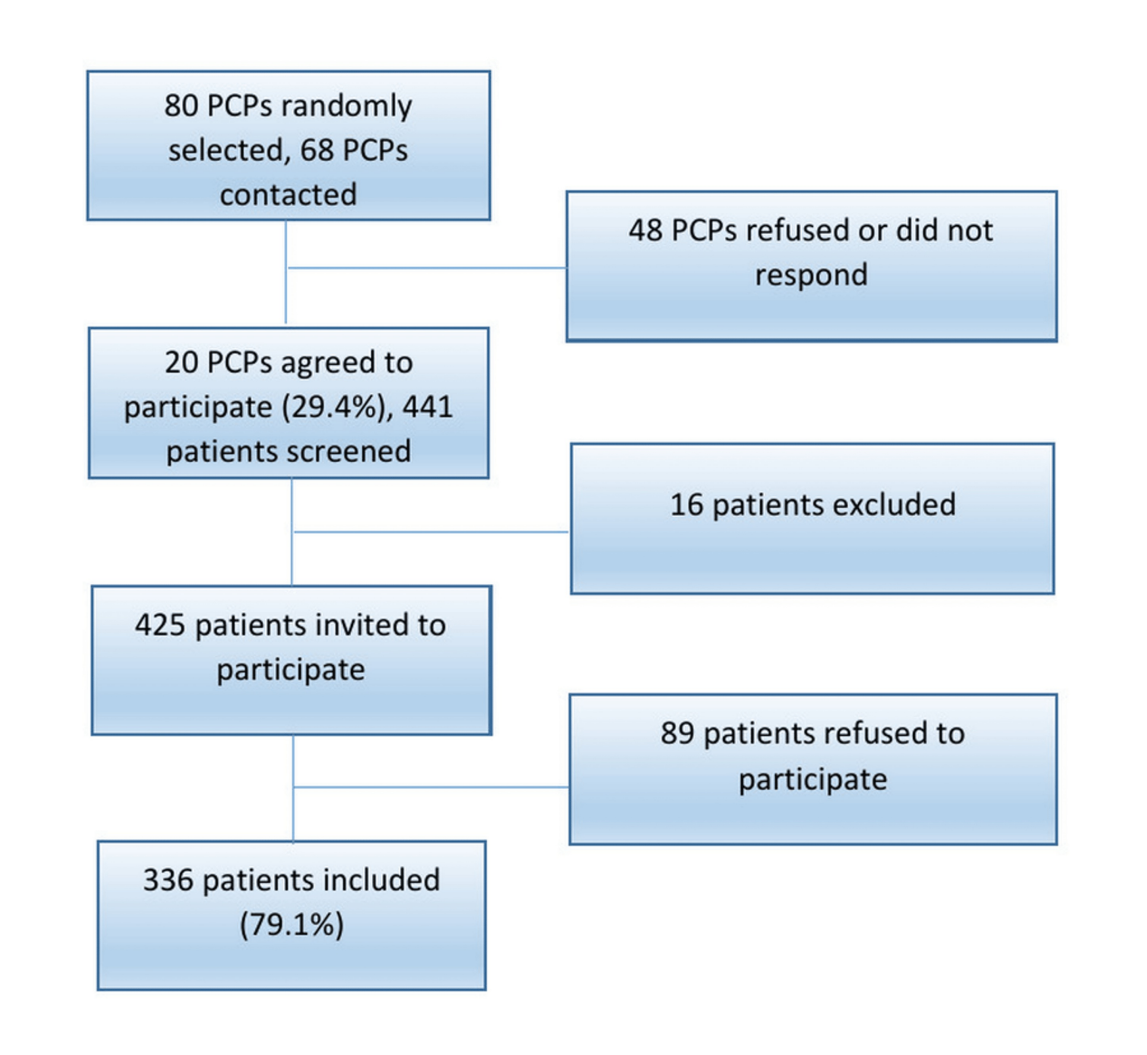
Flowchart of the study. PCP, primary care physician.

Figure 1 illustrates the recruitment process. A total of 68 primary care physicians (PCPs) were randomly selected from a professional register of physicians practicing in the canton of Geneva and were contacted to participate in the study, of whom 20 agreed to recruit patients from their practices. Participants were non-urgent, French-speaking, consecutive adult patients who were able to understand the study and provide written informed consent. All eligible patients who presented during the recruitment period were invited to participate. Patients were excluded if they were under 18 years old, seen in an emergency situation, unable to read and understand French, or unable to provide informed consent.

Patient recruitment was conducted by two research assistants. Participating patients were informed about the study through a poster and were recruited in the waiting room between January 2024 and May 2024. The research assistant verified that participants met the inclusion criteria and were available to answer any questions. Both research assistants underwent standardized training to ensure consistency in patient recruitment. This training encompassed study presentation (guidelines on effectively communicating the study's purpose and procedures to potential participants), information sheet explanation (detailed instructions on how to explain the information sheet to ensure participants' comprehensive understanding), and ethical protocols (emphasis on adhering to ethical standards, including obtaining informed consent and maintaining participant confidentiality). Of the 425 eligible patients, 336 agreed to participate, resulting in a response rate of 79%.

Data collection

Data were collected during routine consultations while patients were in the waiting room. The questionnaire gathered demographic information (gender, age, place of residence, and occupation), self-reported meat consumption, and levels of meat attachment.

We developed the meat consumption questionnaire based on the meat and fish section of the Food Frequency Questionnaire designed for the European Prospective Investigation into Cancer and Nutrition (EPIC) study [14]. This questionnaire specifically assessed self-reported consumption patterns for poultry, beef, veal, and pork. However, seafood was excluded from this study due to cultural and psychological distinctions that often separate it from land-based meats. Including seafood could introduce variability that might confound the assessment of attachment to terrestrial meats. Therefore, this study focuses specifically on land-based meats to maintain clarity in measuring meat attachment. The meat consumption questionnaire is available in Appendix A.

Meat attachment levels were measured using the French version of the MAQ. In this study, the MAQ was self-administered by participants while waiting for their medical consultation. Given its straightforward Likert-scale format, it does not require a trained interviewer for administration.

Meat attachment questionnaire (MAQ)

The 16-item Meat Attachment Questionnaire (MAQ-16) assesses individuals' attachment to meat consumption across four key dimensions: hedonism, affinity, entitlement, and dependence [6]. Responses are provided on a 5-point Likert scale, with mean scores ranging from 1 to 5 and total scores ranging from 16 to 80. Items #4, #6, #9, #13, and #14 are reverse-coded. A higher overall score reflects a stronger attachment to meat.

For the French adaptation, we introduced a modified version with 17 items (MAQf-17), in which item #15 was split into two distinct statements: #15a ("Eating meat is a natural practice") and #15b ("Eating meat is an unquestionable practice"). This 17-item version, which was recently validated by our research team [12], is available in Appendix B. For this study, we used the 16-item version of the MAQ (MAQf-16), excluding item #15b, rather than the 17-item version (MAQf-17), for two main reasons. First, this ensures consistency with other validated versions of the questionnaire, particularly the original English and Portuguese versions, which also contain 16 items [6]. Second, we found that the 16-item and 17-item versions were perfectly correlated (Spearman’s rank correlation coefficient = 1.00, p-value < 0.001), meaning that the additional 17th item ("Eating meat is an unquestionable practice") did not provide further discriminatory power or additional insights.

Ethical considerations

The study was conducted in accordance with the Declaration of Helsinki. All participants provided written informed consent prior to inclusion. The study was approved by the Research Ethics Committee of the University College of General Practice at Claude Bernard University (Project ID: IRB 2023-01-03-01) and was granted a waiver from the Cantonal Research Ethics Commission of Geneva (Project ID: 2023-01941).

Statistical analysis

Descriptive statistics were used to summarize patients' characteristics, meat consumption patterns, and MAQ mean and total scores. The MAQ mean and total scores were found to follow a normal distribution. We assessed normality using both graphical and numerical methods, including the Shapiro-Wilk and Shapiro-Francia tests. The results from both tests were concordant, supporting the normality of the data distribution. We tested for homogeneity of variances using Bartlett's test. Additionally, we examined the normality and homoscedasticity of the residuals.

Numerical variables were reported as means with standard deviations (SD), except for age, which was summarized using medians with interquartile ranges (IQR) due to its non-normal distribution. Categorical variables were presented as frequencies and percentages. Independent t-tests, ANOVA, and chi-square tests were used to assess differences in MAQ mean/total scores and meat consumption by gender and age group. Multivariable linear and logistic regression models, adjusted for age, gender, residence, occupation, and accounting for intra-cluster correlation within practices, were used to examine adjusted associations. ANOVA and multivariable linear regression analyses were performed to examine the unadjusted and adjusted relationships between MAQ scores and levels of meat consumption. Statistical significance was set at p-value < 0.05. All analyses were conducted using Stata 15.1 (StataCorp LLC, College Station, TX).

## Results

Participants’ characteristics

The study included 336 participants, of whom 204 (61%) were female individuals. The median age of participants was 53 years (IQR 28), with ages ranging from 18 to 94 years. The majority of participants (85%) resided in the canton of Geneva. In terms of occupation, 33% were employed, 27% were retired, 14% were unemployed, and 13% held managerial or professional occupations (Table 1).

**Table 1:** Patients’ characteristics. IQR, interquartile range.

Characteristic	N (%)	Median (IQR)	Min-max
Gender (n=333)	-	-	-
Female	204 (61.3)	-	-
Male	127 (38.1)	-	-
Other	2 (0.6)	-	-
Age (years) (n=330)	-	53 (28)	18-94
<40	88 (26.7)	-	-
40-59	119 (36.0)	-	-
≥ 60	123 (37.3)	-	-
Residence (n=325)	-	-	-
Geneva	277 (85.2)	-	-
Vaud	22 (6.8)	-	-
France	18 (5.5)	-	-
Other	8 (2.5)	-	-
Occupation (n=329)	-	-	-
Employee	109 (33.1)	-	-
Retired	89 (27.0)	-	-
Unemployed	46 (14.0)	-	-
Managerial and professional occupations	43 (13.1)	-	-
Intermediate-level profession	24 (7.3)	-	-
Other	18 (5.5)	-	-

Meat attachment mean and total scores

The overall mean score was 3.3 (SD = 0.7), with male participants reporting higher attachment to meat (mean = 3.5) compared to female participants (mean = 3.2) (p-value < 0.001). The adjusted multivariable analysis confirmed these differences (adjusted p-value < 0.001). MAQ scores did not significantly differ by age group (<40 years, 40-59 years, ≥60 years). Similarly, total scores (mean = 52.0, SD = 11.7) were higher among male participants (mean = 55.6) than female participants (mean = 49.9) (p-value < 0.001), with these differences remaining significant after adjustment (adjusted p-value < 0.001) (Table 2). Table 3 shows the unadjusted and adjusted predicted differences in mean and total scores by gender and age group. The adjusted difference for men compared to women in mean and total scores was 0.4 (95%CI: 0.2-0.6) and 6.3 (95%CI: 3.4-9.1), respectively, with adjusted p-values <0.001.

**Table 2:** MAQ mean and total scores, and levels of poultry, beef, veal, and pork consumption, overall and stratified by gender and age group. Threshold for statistical significance: p-value < 0.05. ^1^ T-tests for MAQ mean and total scores, chi-square tests for meat consumption. ^2^ Multivariable linear regressions for MAQ mean and total scores, multivariable logistic regressions for meat consumption (two categories: < 1 time/week and ≥ 1 time/week); models adjusted for age group, gender, residence, occupation, and intra-cluster correlation within practices. ^3^ ANOVA for MAQ mean and total scores, chi-square tests for meat consumption. ^4^ Multivariable linear regressions for MAQ mean and total scores, multivariable logistic regressions for meat consumption (two categories: < 1 time/week and ≥ 1 time/week); models adjusted for age group, gender, residence, occupation, and intra-cluster correlation within practices. MAQ, Meat Attachment Questionnaire.

Variable	Overall	Female	Male	Unadjusted p-value^1^	Adjusted p-value^2^	<40y	40-59y	≥60y	Unadjusted p-value^3^	Adjusted p-value^4^
MAQ mean score (SD) (n=321)	3.3 (0.7)	3.2 (0.7)	3.5 (0.7)	<0.001	<0.001	3.4 (0.7)	3.3 (0.8)	3.3 (0.7)	0.42	0.54
MAQ total score (SD) (n=321)	52.0 (11.7)	49.9 (11.7)	55.6 (10.7)	<0.001	<0.001	53.4 (11.9)	51.8 (12.2)	50.9 (10.9)	0.32	0.46
Poultry consumption, n (%) (n=318)	-	-	-	0.07	0.02	-	-	-	<0.001	0.07
Never	29 (9.1)	19 (9.7)	9 (7.7)	-	-	3 (3.6)	9 (7.9)	17 (14.3)	-	-
<1 time/week	77 (24.2)	53 (26.9)	23 (19.5)	-	-	17 (20.2)	22 (19.5)	38 (31.9)	-	-
1 time/week	88 (27.7)	45 (22.8)	43 (36.4)	-	-	20 (23.8)	29 (25.7)	37 (31.1)	-	-
>1 time/week	124 (39.0)	80 (40.6)	43 (36.4)	-	-	44 (52.4)	53 (46.9)	27 (22.7)	-	-
Beef consumption, n (%) (n=318)	-	-	-	0.01	0.001	-	-	-	0.09	0.10
Never	51 (16.0)	39 (19.8)	11 (9.3)	-	-	12 (14.3)	12 (10.6)	27 (22.7)	-	-
<1 time/week	115 (36.2)	77 (39.1)	37 (31.4)	-	-	25 (29.8)	47 (41.6)	43 (36.1)	-	-
1 time/week	96 (30.2)	54 (27.4)	42 (35.6)	-	-	27 (32.1)	34 (30.1)	34 (28.6)	-	-
>1 time/week	56 (17.6)	27 (13.7)	28 (23.7)	-	-	20 (23.8)	20 (17.7)	15 (12.6)	-	-
Veal consumption, n (%) (n=314)	-	-	-	0.03	0.03	-	-	-	0.42	0.39
Never	130 (41.4)	92 (47.4)	37 (31.6)	-	-	41 (49.4)	41 (37.3)	48 (40.3)	-	-
<1 time/week	110 (35.0)	64 (33.0)	45 (38.5)	-	-	24 (28.9)	38 (34.5)	47 (39.5)	-	-
1 time/week	37 (11.8)	18 (9.3)	19 (16.2)	-	-	7 (8.4)	16 (14.6)	13 (10.9)	-	-
>1 time/week	37 (11.8)	20 (10.3)	16 (13.7)	-	-	11 (13.3)	15 (13.6)	11 (9.3)	-	-
Pork consumption, n (%) (n=314)	-	-	-	0.003	0.05	-	-	-	0.86	0.99
Never	138 (44.0)	100 (51.5)	37 (31.6)	-	-	34 (40.5)	48 (43.6)	56 (47.4)	-	-
<1 time/week	98 (31.2)	57 (29.4)	40 (34.2)	-	-	27 (32.1)	32 (29.1)	38 (32.2)	-	-
1 time/week	50 (15.9)	23 (11.9)	27 (23.1)	-	-	15 (17.9)	20 (18.2)	14 (11.9)	-	-
>1 time/week	28 (8.9)	14 (7.2)	13 (11.1)	-	-	8 (9.5)	10 (9.1)	10 (8.5)	-	-

**Table 3:** Predicted differences in MAQ mean and total scores by gender, age group, and levels of poultry, beef, veal, and pork consumption. Threshold for statistical significance: p-value < 0.05. ^1^ Univariable linear regressions; models adjusted for intra-cluster correlation within practices. ^2^ Multivariable linear regressions; models adjusted for age group, gender, residence, occupation, and intra-cluster correlation within practices. MAQ, Meat Attachment Questionnaire.

Variable	Unadjusted difference in MAQ mean score (95%CI)	p-value^1^	Adjusted difference in MAQ mean score (95%CI)	p-value^2^	Unadjusted difference in MAQ total score (95%CI)	p-value^1^	Adjusted difference in MAQ total score (95%CI)	p-value^2^
Gender	-	0.001	-	<0.001	-	0.001	-	<0.001
Female	0	-	0	-	0	-	0	-
Male	0.35 (0.16;0.55)	-	0.38 (0.21;0.56)	-	5.69 (2.54;8.84)	-	6.25 (3.42;9.07)	-
Age group	-	0.63	-	0.54	-	0.54	-	0.46
<40y	0.13 (-0.16;0.43)	-	0.18 (-0.15;0.50)	-	2.51 (-2.18;7.20)	-	3.10 (-2.04;8.25)	-
40-59y	0.04 (-0.21;0.29)	-	0.10 (-0.18;0.39)	-	0.91 (-3.06;4.89)	-	1.88 (-2.72;6.47)	-
≥60y	0	-	0	-	0	-	0	-
Poultry consumption	-	<0.001	-	<0.001	-	<0.001	-	<0.001
Never	0	-	0	-	0	-	0	-
<1 time/week	0.34 (0.06;0.62)	-	0.31 (0.08;0.53)	-	5.70 (1.07;10.33)	-	4.87 (1.05;8.69)	-
1 time/week	0.78 (0.47;1.09)	-	0.66 (0.38;0.95)	-	12.42 (7.25;17.60)	-	10.23 (5.41;15.04)	-
>1 time/week	0.77 (0.49;1.05)	-	0.75 (0.56;0.94)	-	12.40 (7.72;17.08)	-	11.80 (8.51;15.09)	-
Beef consumption	-	<0.001	-	<0.001	-	<0.001	-	<0.001
Never	0	-	0	-	0	-	0	-
<1 time/week	0.57 (0.32;0.82)	-	0.52 (0.28;0.77)	-	8.99 (5.25;12.74)	-	8.17 (4.45;11.89)	-
1 time/week	0.77 (0.48;1.05)	-	0.69 (0.41;0.97)	-	12.48 (7.96;17.00)	-	11.04 (6.65;15.43)	-
>1 time/week	1.16 (0.88;1.44)	-	1.06 (0.79;1.34)	-	18.66 (14.23;23.09)	-	16.93 (12.45;21.42)	-
Veal consumption	-	<0.001	-	<0.001	-	<0.001	-	<0.001
Never	0	-	0	-	0	-	0	-
<1 time/week	0.39 (0.20;0.58)	-	0.35 (0.18;0.51)	-	6.28 (3.19;9.38)	-	5.68 (3.01;8.36)	-
1 time/week	0.45 (0.24;0.67)	-	0.39 (0.17;0.61)	-	7.48 (3.92;11.04)	-	6.39 (2.73;10.06)	-
>1 time/week	0.84 (0.56;1.12)	-	0.77 (0.50;1.03)	-	13.42 (8.90;17.93)	-	12.20 (7.93;16.48)	-
Pork consumption	-	<0.001	-	<0.001	-	<0.001	-	<0.001
Never	0	-	0	-	0	-	0	-
<1 time/week	0.32 (0.16;0.48)	-	0.26 (0.12;0.41)	-	5.44 (2.83;8.05)	-	4.39 (2.07;6.72)	-
1 time/week	0.49 (0.32;0.65)	-	0.40 (0.23;0.57)	-	8.04 (5.30;10.79)	-	6.62 (3.93;9.32)	-
>1 time/week	0.83 (0.46;1.21)	-	0.77 (0.45;1.08)	-	13.53 (7.43;19.63)	-	12.43 (7.32;17.53)	-

Meat consumption patterns

Poultry was the most commonly consumed meat, with 39% of participants reporting consumption more than once per week. Consumption patterns were relatively similar for male and female participants, though males tended to consume more poultry (p-value = 0.02 after adjustment). Beef was consumed less frequently, with only 18% of participants consuming it more than once per week. Male participants consumed more beef than female participants (p-value = 0.01), and this association persisted after adjustment (adjusted p-value = 0.001). Veal and pork consumption was less frequent overall, but male participants reported higher consumption of both meats compared to female participants (veal, adjusted p-value = 0.03; pork, adjusted p-value = 0.05) (Table 2). There was no significant association with age group (adjusted p-values ranged from 0.07 for poultry to 0.99 for pork).

Association between MAQ scores and meat consumption

Higher MAQ scores were consistently associated with greater consumption of all meat types. For poultry, individuals consuming it more than once per week had a mean score of 3.5, compared to 2.7 for those who never consumed poultry (p-value < 0.001), and a total score of 54.6 versus 42.2, respectively (p-value < 0.001). A similar pattern was observed for beef, veal, and pork, with higher MAQ scores associated with more frequent consumption (p-values < 0.001 for all comparisons). These associations remained significant in multivariable analyses (Table 4). Table 3 shows the unadjusted and adjusted predicted differences in mean and total scores by levels of poultry, beef, veal, and pork consumption. For example, the adjusted difference in mean and total scores for those consuming poultry more than once a week compared to those who never consumed poultry was 0.8 (95%CI: 0.6-0.9) and 11.8 (95%CI: 8.5-15.1), respectively, with adjusted p-values <0.001.

**Table 4:** Association between MAQ mean and total scores, and levels of poultry, beef, veal, and pork consumption. Threshold for statistical significance: p-value < 0.05. ^1^ ANOVA. ^2^ Multivariable linear regressions; models adjusted for age group, gender, residence, occupation, and intra-cluster correlation within practices. MAQ, Meat Attachment Questionnaire.

Variable	N	MAQ mean score (SD)	Unadjusted p-value^1^	Adjusted p-value^2^	MAQ total score (SD)	Unadjusted p-value^1^	Adjusted p-value^2^
Poultry consumption	-	-	<0.001	<0.001	-	<0.001	<0.001
Never	27	2.7 (0.7)	-	-	42.2 (11.1)	-	-
<1 time/week	77	3.0 (0.7)	-	-	47.9 (10.7)	-	-
1 time/week	87	3.5 (0.7)	-	-	54.6 (11.3)	-	-
>1 time/week	118	3.5 (0.7)	-	-	54.6 (11.1)	-	-
Beef consumption	-	-	<0.001	<0.001	-	<0.001	<0.001
Never	49	2.7 (0.7)	-	-	41.5 (11.7)	-	-
<1 time/week	111	3.2 (0.6)	-	-	50.5 (9.4)	-	-
1 time/week	95	3.4 (0.7)	-	-	54.0 (11.0)	-	-
>1 time/week	54	3.8 (0.6)	-	-	60.2 (9.9)	-	-
Veal consumption	-	-	<0.001	<0.001	-	<0.001	<0.001
Never	127	3.0 (0.8)	-	-	47.3 (12.0)	-	-
<1 time/week	108	3.4 (0.6)	-	-	53.6 (10.0)	-	-
1 time/week	36	3.5 (0.6)	-	-	54.8 (9.9)	-	-
>1 time/week	35	3.9 (0.7)	-	-	60.7 (10.5)	-	-
Pork consumption	-	-	<0.001	<0.001	-	<0.001	<0.001
Never	133	3.0 (0.8)	-	-	47.6 (12.0)	-	-
<1 time/week	96	3.4 (0.6)	-	-	53.1 (10.0)	-	-
1 time/week	50	3.5 (0.7)	-	-	55.7 (10.6)	-	-
>1 time/week	28	3.9 (0.6)	-	-	61.1 (9.6)	-	-

## Discussion

Main finding

In this cross-sectional study carried out among Swiss primary care patients, we found that meat attachment, as measured by the MAQ, was significantly associated with higher consumption of all meat types examined (poultry, beef, veal, and pork). Additionally, we observed that men had a stronger attachment to meat and consumed more meat than women.

Comparison with existing literature

Our findings align with previous research demonstrating gender differences in both meat attachment and consumption, with men consistently reporting stronger preferences for meat and higher overall meat intake compared to women [6,15-19]. This phenomenon has been attributed to sociocultural norms that associate masculinity with meat-eating and femininity with plant-based diets [17,18,20,21]. For instance, a study involving Australian and English men found that traditional masculine beliefs related to strength and power were linked to higher red meat consumption [17].

Similar studies have highlighted the psychological and emotional factors that influence meat consumption, suggesting that these attachments may act as barriers to adopting more sustainable dietary patterns [3,22-24]. The concept of 'carnism' (the invisible belief system that conditions people to eat certain animals) plays a crucial role in this context [23]. This belief system leads to the categorization of animals as edible or inedible, influencing perceptions of their sentience and reducing empathy towards them. Additionally, the 'meat paradox' describes the conflict between people's affection for animals and their meat consumption habits, leading to cognitive dissonance [22]. To resolve this dissonance, individuals may employ strategies such as justifying meat consumption as natural, normal, necessary, or nice-the "Four Ns" [24].

While prior literature has identified age-related differences in food preferences [25], our study did not observe significant differences in meat attachment or consumption by age group, which could suggest evolving attitudes toward meat across generations. Notably, recent trends indicate a slight increase in the number of households avoiding meat, suggesting shifting preferences among younger populations [26]. Furthermore, initiatives like Veganuary have led meat-eaters to develop a stronger aversion to meat and identify less as meat-eaters after participating, indicating a potential shift in attitudes [27].

Understanding the nuances of meat attachment and plant-based diet motivations is crucial. While lower meat attachment is often associated with a greater willingness to adopt a plant-based diet, these constructs can function independently. For instance, individuals with low meat attachment may not have strong motivations for plant-based eating, as their dietary choices could instead be driven by factors such as habit, availability, or convenience rather than ethical or health concerns. Conversely, those motivated to follow a plant-based diet may still retain some attachment to meat due to cultural or sensory preferences. Prior studies have demonstrated that individuals with high meat attachment scores are less likely to engage with messages promoting plant-based diets, as their attachment acts as a moderator of dietary change [6,12,28]. Moreover, meat-eaters often report negative emotions towards plant-based consumers, such as fear, envy, contempt, and anger, which could further hinder the adoption of plant-based diets [29]. Understanding these nuances may help tailor interventions aimed at promoting dietary shifts by addressing the psychological dimensions influencing both meat consumption and plant-based motivations.

This study contributes to the validation of the MAQ by demonstrating a strong association between meat attachment scores and actual meat consumption, reinforcing its predictive value in dietary research. While gender differences in meat consumption are well-documented, our findings confirm these relationships within a primary care setting, providing further evidence of their consistency across different populations. This aligns with previous research showing that meat attachment acts as a psychological barrier to plant-based dietary transitions [6,12]. Furthermore, the MAQ was found to predict resistance to reducing meat consumption even after controlling for attitudes toward meat and subjective norms [30]. These findings support the reliability and predictive validity of the MAQ, confirming its relevance as a tool for measuring meat attachment and resistance to dietary change.

International relevance

Given the global push towards reducing meat consumption for both health and environmental sustainability, our findings are internationally relevant. Meat consumption has been linked to various health risks, including cardiovascular diseases [4] and certain cancers [5], as well as contributing significantly to global greenhouse gas emissions [31]. Identifying psychological factors, such as meat attachment, that drive consumption could help inform international public health and environmental strategies. Our findings may be particularly relevant in Western countries, where meat consumption is culturally ingrained and where efforts to promote plant-based diets face significant resistance.

However, although excessive consumption of red and processed meat has been associated with certain health risks, meat remains a valuable source of essential nutrients, particularly for vulnerable populations such as pregnant women, infants, and older adults [32,33]. Nutritional recommendations should focus on optimizing meat intake to balance its benefits and potential risks, rather than advocating for a complete reduction.

Clinical implications

From a clinical perspective, understanding various dimensions of a patient's emotional attachment to meat can aid healthcare professionals in tailoring dietary advice. Nutrition counseling for patients with higher MAQ scores could benefit from focusing not only on the health risks of excessive meat consumption but also on addressing the emotional and psychological barriers to reducing meat intake. Clinicians may consider employing motivational interviewing (MI) or cognitive-behavioral therapy (CBT) to help patients modify their dietary behaviors, especially for those at risk of diet-related diseases. MI is a client-centered counseling approach designed to enhance intrinsic motivation for change by exploring and resolving ambivalence [34]. Studies have demonstrated its effectiveness in modifying various health behaviors, including dietary habits [35,36]. For instance, MI has been shown to successfully reduce the intake of saturated fats and increase fruit and vegetable consumption, leading to improved cardiovascular health outcomes [36]. CBT focuses on identifying and restructuring maladaptive thought patterns and behaviors. It has been effectively applied to treat eating disorders and facilitate healthier eating behaviors [37]. Additionally, targeting patients with a greater awareness of environmental issues could help raise awareness about the environmental impacts of meat consumption, appealing to values beyond personal health, supporting a more holistic approach to dietary change.

In the context of primary care medicine, these strategies are particularly important as PCPs often serve as the first point of contact for patients seeking health advice. They have the opportunity to build long-term relationships with patients, allowing for the ongoing assessment of dietary habits and emotional attachments over time. By integrating discussions about meat attachment into routine consultations, PCPs can create a supportive environment that encourages open dialogue about dietary choices. Furthermore, primary care settings are ideal for implementing community-based interventions that promote plant-based diets, combining individual counseling with broader educational initiatives. This comprehensive approach not only addresses the immediate health concerns of patients but also fosters a greater understanding of the health and environmental implications of their dietary choices, ultimately empowering patients to make informed and sustainable decisions regarding their meat consumption.

Methodological considerations and future research

This study was strengthened by its relatively large sample size and inclusion of diverse patients from primary care practices. However, future research should explore whether these findings can be replicated in other settings or populations, particularly in non-Western contexts where meat consumption patterns may differ. It would also be beneficial to investigate interventions designed to reduce meat attachment and whether these lead to meaningful changes in consumption. Longitudinal studies could provide further insights into the temporal relationship between psychological attachment to meat and dietary behavior change.

Moreover, given that the MAQ is correlated with the willingness to decrease meat consumption, it could serve as a valuable tool for assessing the impact of interventions aimed at reducing meat intake. Future studies should also examine the stability of the MAQ over time to ensure that it remains a reliable measure of meat attachment. Additionally, it is crucial to investigate the stability of the relationship between MAQ scores and actual meat consumption, which would enhance our understanding of how these factors interact over time and inform the development of effective dietary interventions.

Finally, the relationship between meat consumption and health is complex and influenced by multiple dietary and lifestyle factors. While some systematic reviews indicate correlations between high meat intake and chronic disease risk, others suggest that these associations may be confounded by variables such as overall diet quality and physical activity [4,32,38]. Future research should aim to clarify these effects to provide clearer dietary recommendations.

Limitations

This study has several limitations. First, our study was conducted in a primary care setting, where individuals with existing health conditions may be overrepresented compared to the general population. This could potentially influence meat consumption patterns, as dietary behaviors may differ among those managing chronic illnesses or receiving nutritional counseling. Future studies should investigate whether similar associations between meat attachment and consumption are observed in broader population-based samples. Second, women and retirees were overrepresented in our sample. Since gender and age may influence meat consumption patterns, this demographic imbalance may have affected our results. Future studies should aim for a more representative sample to improve the generalizability of the findings to the broader population. Third, the cross-sectional design prevents us from establishing causal relationships between meat attachment and consumption patterns, limiting our ability to draw inferences about the directionality of this association. Fourth, the study relied on self-reported data, which may introduce recall bias or social desirability bias, particularly in reporting meat consumption frequencies. This is an important consideration, as self-reported data is susceptible to misreporting, and participants may underreport behaviors perceived as undesirable. Fifth, the sample was drawn from a specific geographic region (Geneva), which limits the external validity of the findings to other regions or countries with different cultural attitudes towards meat consumption. The cultural homogeneity of our sample may have impacted the diversity of meat consumption behaviors observed. Additionally, other factors such as comorbid conditions, ethical or religious beliefs, and their potential confounding effects on meat attachment and consumption were not assessed in this study, but they may also influence the results. These factors should be considered in future research to better understand the broader context of meat consumption behaviors.

## Conclusions

This study reinforces the strong association between meat attachment and higher levels of meat consumption, highlighting its role as a psychological determinant of dietary behavior. Our findings contribute to the validation of the MAQ in French and demonstrate its relevance in dietary research. However, the cross-sectional design of our study limits conclusions about causality or directionality. Despite this, the observed association suggests that emotional attachment to meat influences consumption patterns. These insights have implications for public health strategies focused on promoting dietary changes. Tailored interventions that address the emotional aspects of meat consumption could improve dietary outcomes. Future research should explore how MAQ-based assessments can be integrated into dietary counseling and behavior change strategies.

## Appendices

Appendix A 

**Table 5:** Meat consumption questionnaire. Based on: [14]

Please check your average consumption over the past 12 months	Never or less than once a month	1–3 times per month	Once a week	2–4 times per week	5–6 times per week	Once a day	2–3 times per day	4–5 times per day	6 or more times per day
1/ Beef: ground beef, beef roast, beef steak, beef ribs, beef bourguignon, braised beef, pot-au-feu, etc.	☐	☐	☐	☐	☐	☐	☐	☐	☐
2/ Veal: veal escalope, veal roast, veal chop, veal stew, blanquette, osso-buco, etc.	☐	☐	☐	☐	☐	☐	☐	☐	☐
3/ Pork: pork chops, pork roast, pork loin, spare ribs, pork tenderloin, etc.	☐	☐	☐	☐	☐	☐	☐	☐	☐
4/ Lamb: lamb chops, lamb shoulder, lamb leg, lamb stew, navarin, etc.	☐	☐	☐	☐	☐	☐	☐	☐	☐
5/ Poultry: turkey, chicken, duck, etc.	☐	☐	☐	☐	☐	☐	☐	☐	☐

Appendix B 

**Table 6:** Meat attachment questionnaire. Source: [12]

Please check the answer that best corresponds to you for each of the questions	Strongly disagree	Disagree	Neutral	Agree	Strongly agree
1/ To eat meat is one of the good pleasures in life	☐	☐	☐	☐	☐
2/ Meat is irreplaceable in my diet	☐	☐	☐	☐	☐
3/ According to our position in the food chain, we have the right to eat meat	☐	☐	☐	☐	☐
4/ I feel bad when I think of eating meat	☐	☐	☐	☐	☐
5/ I love meals with meat	☐	☐	☐	☐	☐
6/ To eat meat is disrespectful towards life and the environment	☐	☐	☐	☐	☐
7/ To eat meat is an unquestionable right of every person	☐	☐	☐	☐	☐
8/ A good steak is without comparison	☐	☐	☐	☐	☐
9/ I would feel fine with a meatless diet	☐	☐	☐	☐	☐
10/ I’m a big fan of meat	☐	☐	☐	☐	☐
11/ If I couldn’t eat meat I would feel weak	☐	☐	☐	☐	☐
12/ If I was forced to stop eating meat I would feel sad	☐	☐	☐	☐	☐
13/ Meat reminds me of diseases	☐	☐	☐	☐	☐
14/ By eating meat I’m reminded of the death and suffering of animals	☐	☐	☐	☐	☐
15a/ Eating meat is a natural practice	☐	☐	☐	☐	☐
15b/ Eating meat is a undisputable practice	☐	☐	☐	☐	☐
16/ I don’t picture myself without eating meat regularly	☐	☐	☐	☐	☐
